# Supplementary figures and images for: Estimating and abstracting the 3D structure of feline bones using neural networks on X-ray (2D) images (part 1 of 2)
Source: Commun Biol. 2020 Jun 30;3:337. doi: 10.1038/s42003-020-1057-3 (PMC7326932; doi:10.1038/s42003-020-1057-3)

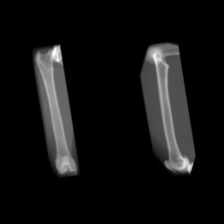

Supplement: Supplementary file 1 — Supplementary Data 1 [file 42003_2020_1057_MOESM1_ESM.zip › 24_bones_classifier_train_valid_224x224x3/train/0/0_APML.dcm_rotation103,9_energy146.jpg]

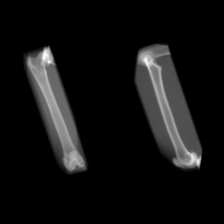

Supplement: Supplementary file 1 — Supplementary Data 1 [file 42003_2020_1057_MOESM1_ESM.zip › 24_bones_classifier_train_valid_224x224x3/train/0/0_APML.dcm_rotation109,18_energy146.jpg]

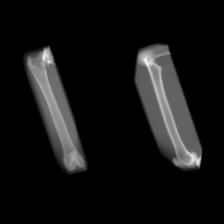

Supplement: Supplementary file 1 — Supplementary Data 1 [file 42003_2020_1057_MOESM1_ESM.zip › 24_bones_classifier_train_valid_224x224x3/train/0/0_APML.dcm_rotation109,18_energy152.jpg]

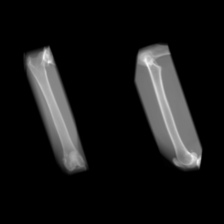

Supplement: Supplementary file 1 — Supplementary Data 1 [file 42003_2020_1057_MOESM1_ESM.zip › 24_bones_classifier_train_valid_224x224x3/train/0/0_APML.dcm_rotation109,18_energy158.jpg]

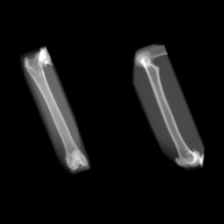

Supplement: Supplementary file 1 — Supplementary Data 1 [file 42003_2020_1057_MOESM1_ESM.zip › 24_bones_classifier_train_valid_224x224x3/train/0/0_APML.dcm_rotation109,21_energy140.jpg]

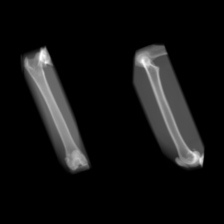

Supplement: Supplementary file 1 — Supplementary Data 1 [file 42003_2020_1057_MOESM1_ESM.zip › 24_bones_classifier_train_valid_224x224x3/train/0/0_APML.dcm_rotation109,21_energy146.jpg]

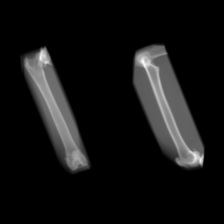

Supplement: Supplementary file 1 — Supplementary Data 1 [file 42003_2020_1057_MOESM1_ESM.zip › 24_bones_classifier_train_valid_224x224x3/train/0/0_APML.dcm_rotation109,21_energy152.jpg]

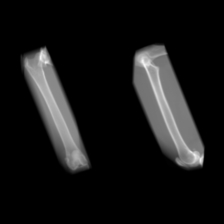

Supplement: Supplementary file 1 — Supplementary Data 1 [file 42003_2020_1057_MOESM1_ESM.zip › 24_bones_classifier_train_valid_224x224x3/train/0/0_APML.dcm_rotation109,21_energy158.jpg]

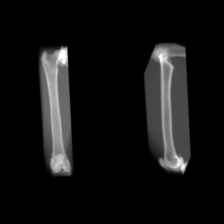

Supplement: Supplementary file 1 — Supplementary Data 1 [file 42003_2020_1057_MOESM1_ESM.zip › 24_bones_classifier_train_valid_224x224x3/train/0/0_APML.dcm_rotation109,3_energy140.jpg]

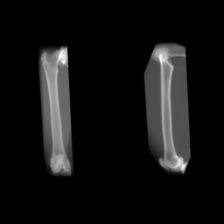

Supplement: Supplementary file 1 — Supplementary Data 1 [file 42003_2020_1057_MOESM1_ESM.zip › 24_bones_classifier_train_valid_224x224x3/train/0/0_APML.dcm_rotation109,3_energy146.jpg]

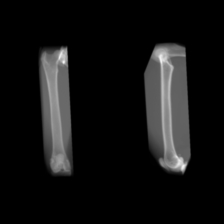

Supplement: Supplementary file 1 — Supplementary Data 1 [file 42003_2020_1057_MOESM1_ESM.zip › 24_bones_classifier_train_valid_224x224x3/train/0/0_APML.dcm_rotation109,3_energy152.jpg]

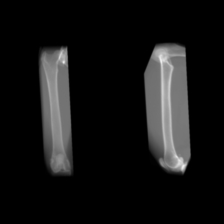

Supplement: Supplementary file 1 — Supplementary Data 1 [file 42003_2020_1057_MOESM1_ESM.zip › 24_bones_classifier_train_valid_224x224x3/train/0/0_APML.dcm_rotation109,3_energy158.jpg]

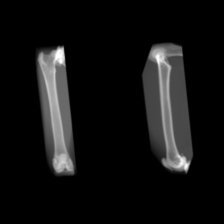

Supplement: Supplementary file 1 — Supplementary Data 1 [file 42003_2020_1057_MOESM1_ESM.zip › 24_bones_classifier_train_valid_224x224x3/train/0/0_APML.dcm_rotation109,6_energy140.jpg]

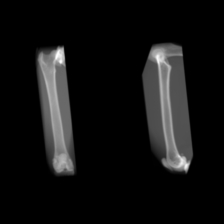

Supplement: Supplementary file 1 — Supplementary Data 1 [file 42003_2020_1057_MOESM1_ESM.zip › 24_bones_classifier_train_valid_224x224x3/train/0/0_APML.dcm_rotation109,6_energy146.jpg]

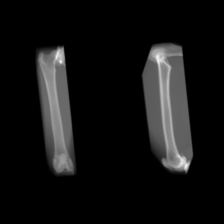

Supplement: Supplementary file 1 — Supplementary Data 1 [file 42003_2020_1057_MOESM1_ESM.zip › 24_bones_classifier_train_valid_224x224x3/train/0/0_APML.dcm_rotation109,6_energy152.jpg]

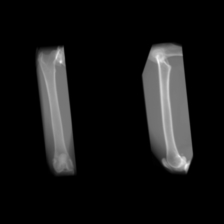

Supplement: Supplementary file 1 — Supplementary Data 1 [file 42003_2020_1057_MOESM1_ESM.zip › 24_bones_classifier_train_valid_224x224x3/train/0/0_APML.dcm_rotation109,6_energy158.jpg]

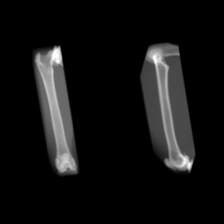

Supplement: Supplementary file 1 — Supplementary Data 1 [file 42003_2020_1057_MOESM1_ESM.zip › 24_bones_classifier_train_valid_224x224x3/train/0/0_APML.dcm_rotation109,9_energy140.jpg]

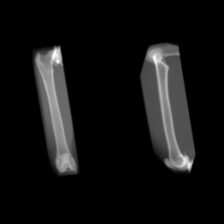

Supplement: Supplementary file 1 — Supplementary Data 1 [file 42003_2020_1057_MOESM1_ESM.zip › 24_bones_classifier_train_valid_224x224x3/train/0/0_APML.dcm_rotation109,9_energy146.jpg]

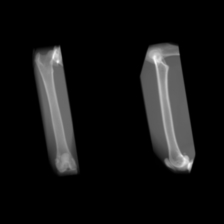

Supplement: Supplementary file 1 — Supplementary Data 1 [file 42003_2020_1057_MOESM1_ESM.zip › 24_bones_classifier_train_valid_224x224x3/train/0/0_APML.dcm_rotation109,9_energy152.jpg]

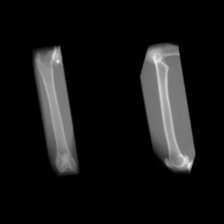

Supplement: Supplementary file 1 — Supplementary Data 1 [file 42003_2020_1057_MOESM1_ESM.zip › 24_bones_classifier_train_valid_224x224x3/train/0/0_APML.dcm_rotation109,9_energy158.jpg]

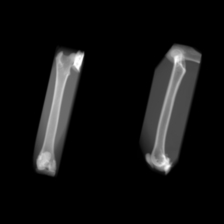

Supplement: Supplementary file 1 — Supplementary Data 1 [file 42003_2020_1057_MOESM1_ESM.zip › 24_bones_classifier_train_valid_224x224x3/train/0/0_APML.dcm_rotation112,-12_energy140.jpg]

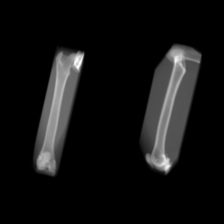

Supplement: Supplementary file 1 — Supplementary Data 1 [file 42003_2020_1057_MOESM1_ESM.zip › 24_bones_classifier_train_valid_224x224x3/train/0/0_APML.dcm_rotation112,-12_energy146.jpg]

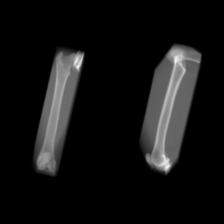

Supplement: Supplementary file 1 — Supplementary Data 1 [file 42003_2020_1057_MOESM1_ESM.zip › 24_bones_classifier_train_valid_224x224x3/train/0/0_APML.dcm_rotation112,-12_energy152.jpg]

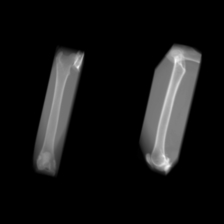

Supplement: Supplementary file 1 — Supplementary Data 1 [file 42003_2020_1057_MOESM1_ESM.zip › 24_bones_classifier_train_valid_224x224x3/train/0/0_APML.dcm_rotation112,-12_energy158.jpg]

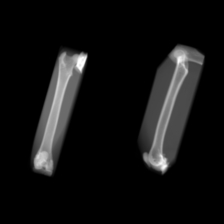

Supplement: Supplementary file 1 — Supplementary Data 1 [file 42003_2020_1057_MOESM1_ESM.zip › 24_bones_classifier_train_valid_224x224x3/train/0/0_APML.dcm_rotation112,-15_energy140.jpg]

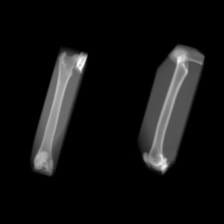

Supplement: Supplementary file 1 — Supplementary Data 1 [file 42003_2020_1057_MOESM1_ESM.zip › 24_bones_classifier_train_valid_224x224x3/train/0/0_APML.dcm_rotation112,-15_energy146.jpg]

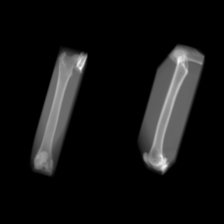

Supplement: Supplementary file 1 — Supplementary Data 1 [file 42003_2020_1057_MOESM1_ESM.zip › 24_bones_classifier_train_valid_224x224x3/train/0/0_APML.dcm_rotation112,-15_energy152.jpg]

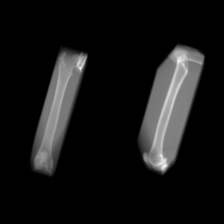

Supplement: Supplementary file 1 — Supplementary Data 1 [file 42003_2020_1057_MOESM1_ESM.zip › 24_bones_classifier_train_valid_224x224x3/train/0/0_APML.dcm_rotation112,-15_energy158.jpg]

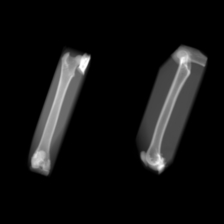

Supplement: Supplementary file 1 — Supplementary Data 1 [file 42003_2020_1057_MOESM1_ESM.zip › 24_bones_classifier_train_valid_224x224x3/train/0/0_APML.dcm_rotation112,-18_energy140.jpg]

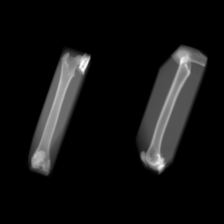

Supplement: Supplementary file 1 — Supplementary Data 1 [file 42003_2020_1057_MOESM1_ESM.zip › 24_bones_classifier_train_valid_224x224x3/train/0/0_APML.dcm_rotation112,-18_energy146.jpg]

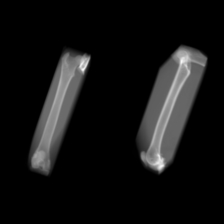

Supplement: Supplementary file 1 — Supplementary Data 1 [file 42003_2020_1057_MOESM1_ESM.zip › 24_bones_classifier_train_valid_224x224x3/train/0/0_APML.dcm_rotation112,-18_energy152.jpg]

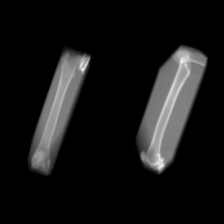

Supplement: Supplementary file 1 — Supplementary Data 1 [file 42003_2020_1057_MOESM1_ESM.zip › 24_bones_classifier_train_valid_224x224x3/train/0/0_APML.dcm_rotation112,-18_energy158.jpg]

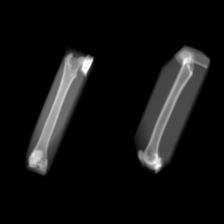

Supplement: Supplementary file 1 — Supplementary Data 1 [file 42003_2020_1057_MOESM1_ESM.zip › 24_bones_classifier_train_valid_224x224x3/train/0/0_APML.dcm_rotation112,-21_energy140.jpg]

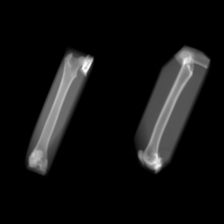

Supplement: Supplementary file 1 — Supplementary Data 1 [file 42003_2020_1057_MOESM1_ESM.zip › 24_bones_classifier_train_valid_224x224x3/train/0/0_APML.dcm_rotation112,-21_energy146.jpg]

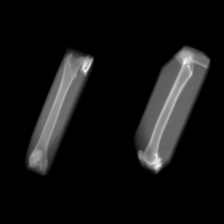

Supplement: Supplementary file 1 — Supplementary Data 1 [file 42003_2020_1057_MOESM1_ESM.zip › 24_bones_classifier_train_valid_224x224x3/train/0/0_APML.dcm_rotation112,-21_energy152.jpg]

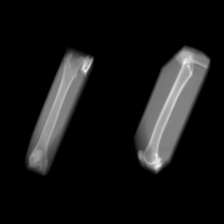

Supplement: Supplementary file 1 — Supplementary Data 1 [file 42003_2020_1057_MOESM1_ESM.zip › 24_bones_classifier_train_valid_224x224x3/train/0/0_APML.dcm_rotation112,-21_energy158.jpg]

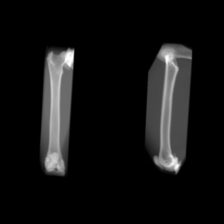

Supplement: Supplementary file 1 — Supplementary Data 1 [file 42003_2020_1057_MOESM1_ESM.zip › 24_bones_classifier_train_valid_224x224x3/train/0/0_APML.dcm_rotation112,-3_energy140.jpg]

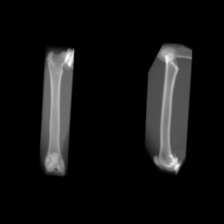

Supplement: Supplementary file 1 — Supplementary Data 1 [file 42003_2020_1057_MOESM1_ESM.zip › 24_bones_classifier_train_valid_224x224x3/train/0/0_APML.dcm_rotation112,-3_energy146.jpg]

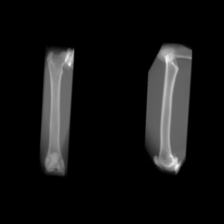

Supplement: Supplementary file 1 — Supplementary Data 1 [file 42003_2020_1057_MOESM1_ESM.zip › 24_bones_classifier_train_valid_224x224x3/train/0/0_APML.dcm_rotation112,-3_energy152.jpg]

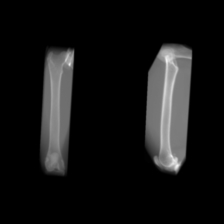

Supplement: Supplementary file 1 — Supplementary Data 1 [file 42003_2020_1057_MOESM1_ESM.zip › 24_bones_classifier_train_valid_224x224x3/train/0/0_APML.dcm_rotation112,-3_energy158.jpg]

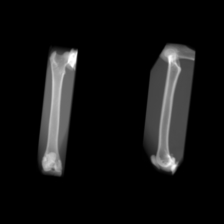

Supplement: Supplementary file 1 — Supplementary Data 1 [file 42003_2020_1057_MOESM1_ESM.zip › 24_bones_classifier_train_valid_224x224x3/train/0/0_APML.dcm_rotation112,-6_energy140.jpg]

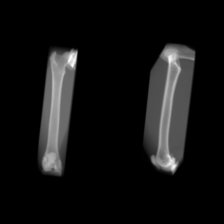

Supplement: Supplementary file 1 — Supplementary Data 1 [file 42003_2020_1057_MOESM1_ESM.zip › 24_bones_classifier_train_valid_224x224x3/train/0/0_APML.dcm_rotation112,-6_energy146.jpg]

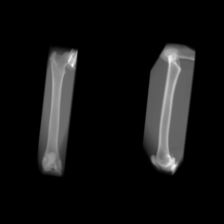

Supplement: Supplementary file 1 — Supplementary Data 1 [file 42003_2020_1057_MOESM1_ESM.zip › 24_bones_classifier_train_valid_224x224x3/train/0/0_APML.dcm_rotation112,-6_energy152.jpg]

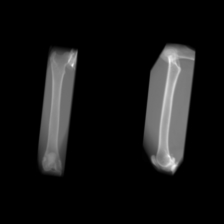

Supplement: Supplementary file 1 — Supplementary Data 1 [file 42003_2020_1057_MOESM1_ESM.zip › 24_bones_classifier_train_valid_224x224x3/train/0/0_APML.dcm_rotation112,-6_energy158.jpg]

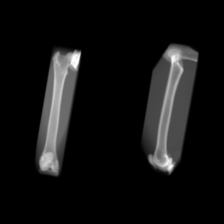

Supplement: Supplementary file 1 — Supplementary Data 1 [file 42003_2020_1057_MOESM1_ESM.zip › 24_bones_classifier_train_valid_224x224x3/train/0/0_APML.dcm_rotation112,-9_energy140.jpg]

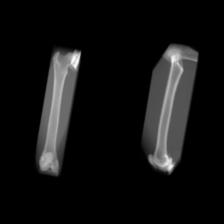

Supplement: Supplementary file 1 — Supplementary Data 1 [file 42003_2020_1057_MOESM1_ESM.zip › 24_bones_classifier_train_valid_224x224x3/train/0/0_APML.dcm_rotation112,-9_energy146.jpg]

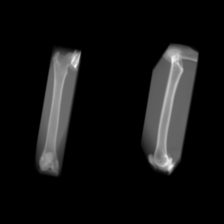

Supplement: Supplementary file 1 — Supplementary Data 1 [file 42003_2020_1057_MOESM1_ESM.zip › 24_bones_classifier_train_valid_224x224x3/train/0/0_APML.dcm_rotation112,-9_energy152.jpg]

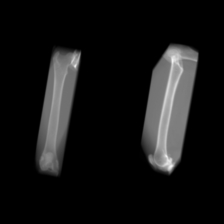

Supplement: Supplementary file 1 — Supplementary Data 1 [file 42003_2020_1057_MOESM1_ESM.zip › 24_bones_classifier_train_valid_224x224x3/train/0/0_APML.dcm_rotation112,-9_energy158.jpg]

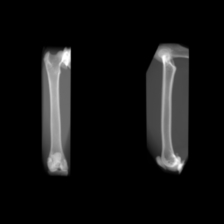

Supplement: Supplementary file 1 — Supplementary Data 1 [file 42003_2020_1057_MOESM1_ESM.zip › 24_bones_classifier_train_valid_224x224x3/train/0/0_APML.dcm_rotation112,0_energy140.jpg]

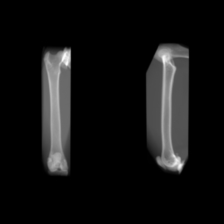

Supplement: Supplementary file 1 — Supplementary Data 1 [file 42003_2020_1057_MOESM1_ESM.zip › 24_bones_classifier_train_valid_224x224x3/train/0/0_APML.dcm_rotation112,0_energy146.jpg]

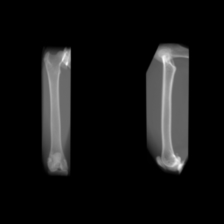

Supplement: Supplementary file 1 — Supplementary Data 1 [file 42003_2020_1057_MOESM1_ESM.zip › 24_bones_classifier_train_valid_224x224x3/train/0/0_APML.dcm_rotation112,0_energy152.jpg]

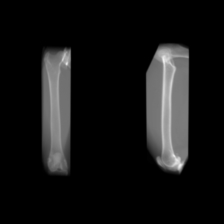

Supplement: Supplementary file 1 — Supplementary Data 1 [file 42003_2020_1057_MOESM1_ESM.zip › 24_bones_classifier_train_valid_224x224x3/train/0/0_APML.dcm_rotation112,0_energy158.jpg]

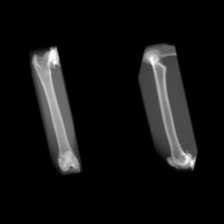

Supplement: Supplementary file 1 — Supplementary Data 1 [file 42003_2020_1057_MOESM1_ESM.zip › 24_bones_classifier_train_valid_224x224x3/train/0/0_APML.dcm_rotation112,12_energy140.jpg]

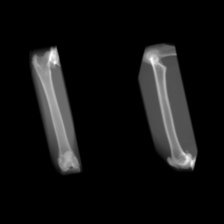

Supplement: Supplementary file 1 — Supplementary Data 1 [file 42003_2020_1057_MOESM1_ESM.zip › 24_bones_classifier_train_valid_224x224x3/train/0/0_APML.dcm_rotation112,12_energy146.jpg]

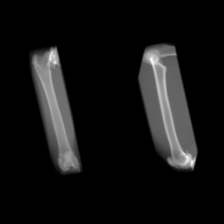

Supplement: Supplementary file 1 — Supplementary Data 1 [file 42003_2020_1057_MOESM1_ESM.zip › 24_bones_classifier_train_valid_224x224x3/train/0/0_APML.dcm_rotation112,12_energy152.jpg]

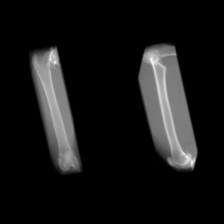

Supplement: Supplementary file 1 — Supplementary Data 1 [file 42003_2020_1057_MOESM1_ESM.zip › 24_bones_classifier_train_valid_224x224x3/train/0/0_APML.dcm_rotation112,12_energy158.jpg]

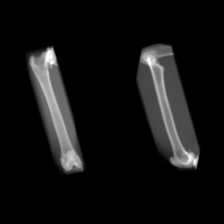

Supplement: Supplementary file 1 — Supplementary Data 1 [file 42003_2020_1057_MOESM1_ESM.zip › 24_bones_classifier_train_valid_224x224x3/train/0/0_APML.dcm_rotation112,15_energy140.jpg]

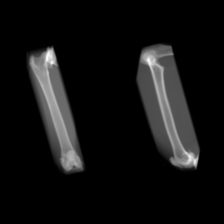

Supplement: Supplementary file 1 — Supplementary Data 1 [file 42003_2020_1057_MOESM1_ESM.zip › 24_bones_classifier_train_valid_224x224x3/train/0/0_APML.dcm_rotation112,15_energy146.jpg]

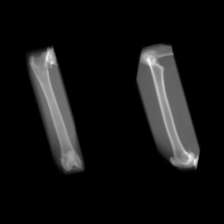

Supplement: Supplementary file 1 — Supplementary Data 1 [file 42003_2020_1057_MOESM1_ESM.zip › 24_bones_classifier_train_valid_224x224x3/train/0/0_APML.dcm_rotation112,15_energy152.jpg]

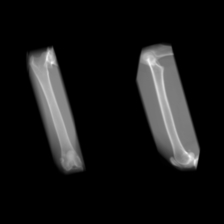

Supplement: Supplementary file 1 — Supplementary Data 1 [file 42003_2020_1057_MOESM1_ESM.zip › 24_bones_classifier_train_valid_224x224x3/train/0/0_APML.dcm_rotation112,15_energy158.jpg]

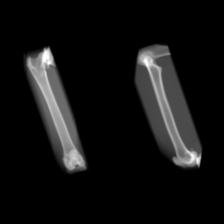

Supplement: Supplementary file 1 — Supplementary Data 1 [file 42003_2020_1057_MOESM1_ESM.zip › 24_bones_classifier_train_valid_224x224x3/train/0/0_APML.dcm_rotation112,18_energy140.jpg]

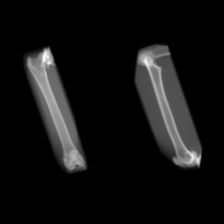

Supplement: Supplementary file 1 — Supplementary Data 1 [file 42003_2020_1057_MOESM1_ESM.zip › 24_bones_classifier_train_valid_224x224x3/train/0/0_APML.dcm_rotation112,18_energy146.jpg]

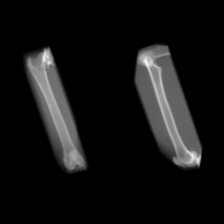

Supplement: Supplementary file 1 — Supplementary Data 1 [file 42003_2020_1057_MOESM1_ESM.zip › 24_bones_classifier_train_valid_224x224x3/train/0/0_APML.dcm_rotation112,18_energy152.jpg]

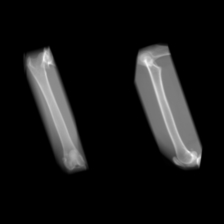

Supplement: Supplementary file 1 — Supplementary Data 1 [file 42003_2020_1057_MOESM1_ESM.zip › 24_bones_classifier_train_valid_224x224x3/train/0/0_APML.dcm_rotation112,18_energy158.jpg]

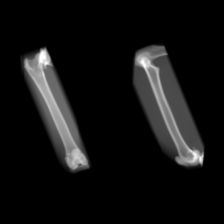

Supplement: Supplementary file 1 — Supplementary Data 1 [file 42003_2020_1057_MOESM1_ESM.zip › 24_bones_classifier_train_valid_224x224x3/train/0/0_APML.dcm_rotation112,21_energy140.jpg]

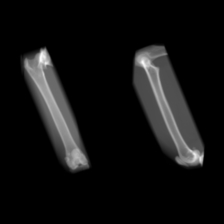

Supplement: Supplementary file 1 — Supplementary Data 1 [file 42003_2020_1057_MOESM1_ESM.zip › 24_bones_classifier_train_valid_224x224x3/train/0/0_APML.dcm_rotation112,21_energy146.jpg]

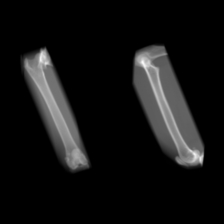

Supplement: Supplementary file 1 — Supplementary Data 1 [file 42003_2020_1057_MOESM1_ESM.zip › 24_bones_classifier_train_valid_224x224x3/train/0/0_APML.dcm_rotation112,21_energy152.jpg]

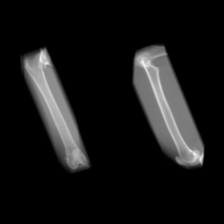

Supplement: Supplementary file 1 — Supplementary Data 1 [file 42003_2020_1057_MOESM1_ESM.zip › 24_bones_classifier_train_valid_224x224x3/train/0/0_APML.dcm_rotation112,21_energy158.jpg]

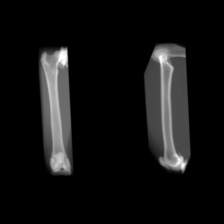

Supplement: Supplementary file 1 — Supplementary Data 1 [file 42003_2020_1057_MOESM1_ESM.zip › 24_bones_classifier_train_valid_224x224x3/train/0/0_APML.dcm_rotation112,3_energy140.jpg]

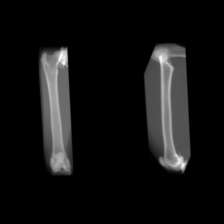

Supplement: Supplementary file 1 — Supplementary Data 1 [file 42003_2020_1057_MOESM1_ESM.zip › 24_bones_classifier_train_valid_224x224x3/train/0/0_APML.dcm_rotation112,3_energy146.jpg]

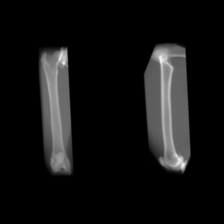

Supplement: Supplementary file 1 — Supplementary Data 1 [file 42003_2020_1057_MOESM1_ESM.zip › 24_bones_classifier_train_valid_224x224x3/train/0/0_APML.dcm_rotation112,3_energy152.jpg]

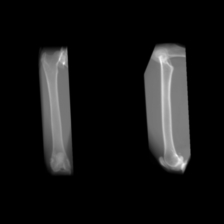

Supplement: Supplementary file 1 — Supplementary Data 1 [file 42003_2020_1057_MOESM1_ESM.zip › 24_bones_classifier_train_valid_224x224x3/train/0/0_APML.dcm_rotation112,3_energy158.jpg]

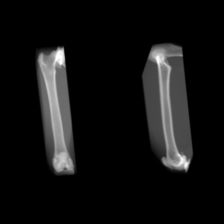

Supplement: Supplementary file 1 — Supplementary Data 1 [file 42003_2020_1057_MOESM1_ESM.zip › 24_bones_classifier_train_valid_224x224x3/train/0/0_APML.dcm_rotation112,6_energy140.jpg]

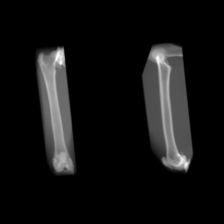

Supplement: Supplementary file 1 — Supplementary Data 1 [file 42003_2020_1057_MOESM1_ESM.zip › 24_bones_classifier_train_valid_224x224x3/train/0/0_APML.dcm_rotation112,6_energy146.jpg]

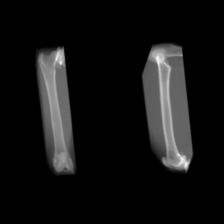

Supplement: Supplementary file 1 — Supplementary Data 1 [file 42003_2020_1057_MOESM1_ESM.zip › 24_bones_classifier_train_valid_224x224x3/train/0/0_APML.dcm_rotation112,6_energy152.jpg]

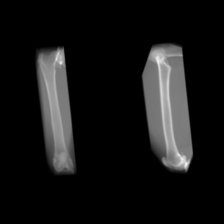

Supplement: Supplementary file 1 — Supplementary Data 1 [file 42003_2020_1057_MOESM1_ESM.zip › 24_bones_classifier_train_valid_224x224x3/train/0/0_APML.dcm_rotation112,6_energy158.jpg]

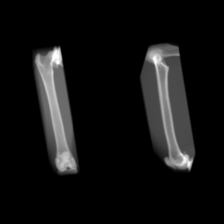

Supplement: Supplementary file 1 — Supplementary Data 1 [file 42003_2020_1057_MOESM1_ESM.zip › 24_bones_classifier_train_valid_224x224x3/train/0/0_APML.dcm_rotation112,9_energy140.jpg]

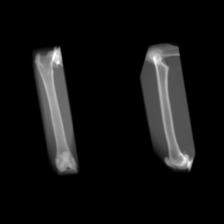

Supplement: Supplementary file 1 — Supplementary Data 1 [file 42003_2020_1057_MOESM1_ESM.zip › 24_bones_classifier_train_valid_224x224x3/train/0/0_APML.dcm_rotation112,9_energy146.jpg]

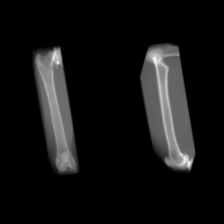

Supplement: Supplementary file 1 — Supplementary Data 1 [file 42003_2020_1057_MOESM1_ESM.zip › 24_bones_classifier_train_valid_224x224x3/train/0/0_APML.dcm_rotation112,9_energy152.jpg]

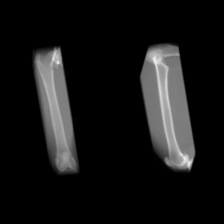

Supplement: Supplementary file 1 — Supplementary Data 1 [file 42003_2020_1057_MOESM1_ESM.zip › 24_bones_classifier_train_valid_224x224x3/train/0/0_APML.dcm_rotation112,9_energy158.jpg]

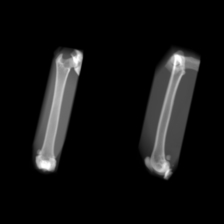

Supplement: Supplementary file 1 — Supplementary Data 1 [file 42003_2020_1057_MOESM1_ESM.zip › 24_bones_classifier_train_valid_224x224x3/train/0/0_APML.dcm_rotation70,-12_energy140.jpg]

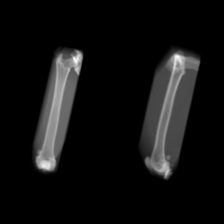

Supplement: Supplementary file 1 — Supplementary Data 1 [file 42003_2020_1057_MOESM1_ESM.zip › 24_bones_classifier_train_valid_224x224x3/train/0/0_APML.dcm_rotation70,-12_energy146.jpg]

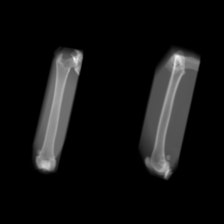

Supplement: Supplementary file 1 — Supplementary Data 1 [file 42003_2020_1057_MOESM1_ESM.zip › 24_bones_classifier_train_valid_224x224x3/train/0/0_APML.dcm_rotation70,-12_energy152.jpg]

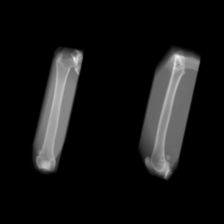

Supplement: Supplementary file 1 — Supplementary Data 1 [file 42003_2020_1057_MOESM1_ESM.zip › 24_bones_classifier_train_valid_224x224x3/train/0/0_APML.dcm_rotation70,-12_energy158.jpg]

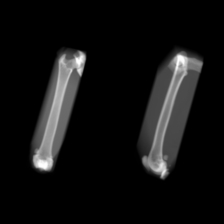

Supplement: Supplementary file 1 — Supplementary Data 1 [file 42003_2020_1057_MOESM1_ESM.zip › 24_bones_classifier_train_valid_224x224x3/train/0/0_APML.dcm_rotation70,-15_energy140.jpg]

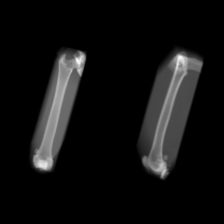

Supplement: Supplementary file 1 — Supplementary Data 1 [file 42003_2020_1057_MOESM1_ESM.zip › 24_bones_classifier_train_valid_224x224x3/train/0/0_APML.dcm_rotation70,-15_energy146.jpg]

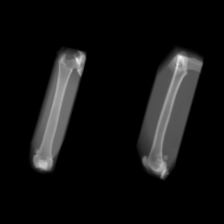

Supplement: Supplementary file 1 — Supplementary Data 1 [file 42003_2020_1057_MOESM1_ESM.zip › 24_bones_classifier_train_valid_224x224x3/train/0/0_APML.dcm_rotation70,-15_energy152.jpg]

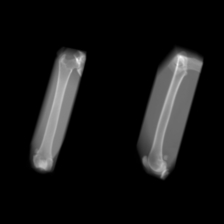

Supplement: Supplementary file 1 — Supplementary Data 1 [file 42003_2020_1057_MOESM1_ESM.zip › 24_bones_classifier_train_valid_224x224x3/train/0/0_APML.dcm_rotation70,-15_energy158.jpg]

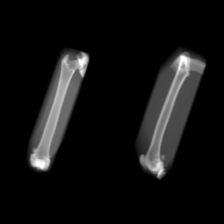

Supplement: Supplementary file 1 — Supplementary Data 1 [file 42003_2020_1057_MOESM1_ESM.zip › 24_bones_classifier_train_valid_224x224x3/train/0/0_APML.dcm_rotation70,-18_energy140.jpg]

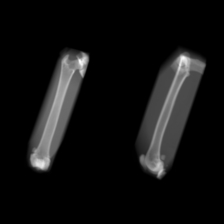

Supplement: Supplementary file 1 — Supplementary Data 1 [file 42003_2020_1057_MOESM1_ESM.zip › 24_bones_classifier_train_valid_224x224x3/train/0/0_APML.dcm_rotation70,-18_energy146.jpg]

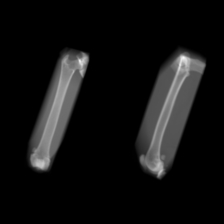

Supplement: Supplementary file 1 — Supplementary Data 1 [file 42003_2020_1057_MOESM1_ESM.zip › 24_bones_classifier_train_valid_224x224x3/train/0/0_APML.dcm_rotation70,-18_energy152.jpg]

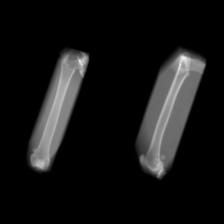

Supplement: Supplementary file 1 — Supplementary Data 1 [file 42003_2020_1057_MOESM1_ESM.zip › 24_bones_classifier_train_valid_224x224x3/train/0/0_APML.dcm_rotation70,-18_energy158.jpg]

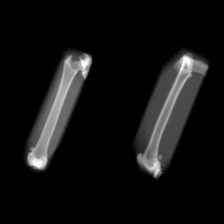

Supplement: Supplementary file 1 — Supplementary Data 1 [file 42003_2020_1057_MOESM1_ESM.zip › 24_bones_classifier_train_valid_224x224x3/train/0/0_APML.dcm_rotation70,-21_energy140.jpg]

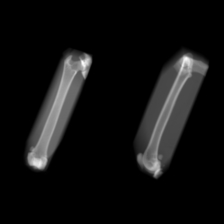

Supplement: Supplementary file 1 — Supplementary Data 1 [file 42003_2020_1057_MOESM1_ESM.zip › 24_bones_classifier_train_valid_224x224x3/train/0/0_APML.dcm_rotation70,-21_energy146.jpg]

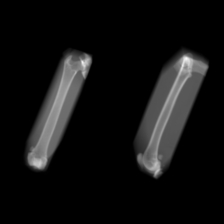

Supplement: Supplementary file 1 — Supplementary Data 1 [file 42003_2020_1057_MOESM1_ESM.zip › 24_bones_classifier_train_valid_224x224x3/train/0/0_APML.dcm_rotation70,-21_energy152.jpg]

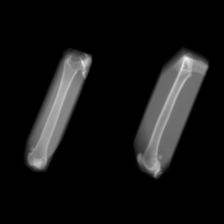

Supplement: Supplementary file 1 — Supplementary Data 1 [file 42003_2020_1057_MOESM1_ESM.zip › 24_bones_classifier_train_valid_224x224x3/train/0/0_APML.dcm_rotation70,-21_energy158.jpg]

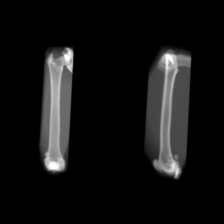

Supplement: Supplementary file 1 — Supplementary Data 1 [file 42003_2020_1057_MOESM1_ESM.zip › 24_bones_classifier_train_valid_224x224x3/train/0/0_APML.dcm_rotation70,-3_energy140.jpg]

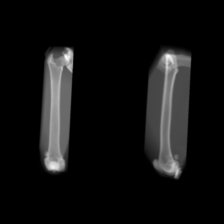

Supplement: Supplementary file 1 — Supplementary Data 1 [file 42003_2020_1057_MOESM1_ESM.zip › 24_bones_classifier_train_valid_224x224x3/train/0/0_APML.dcm_rotation70,-3_energy146.jpg]

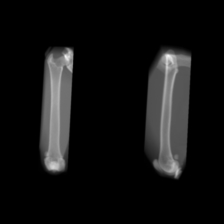

Supplement: Supplementary file 1 — Supplementary Data 1 [file 42003_2020_1057_MOESM1_ESM.zip › 24_bones_classifier_train_valid_224x224x3/train/0/0_APML.dcm_rotation70,-3_energy152.jpg]

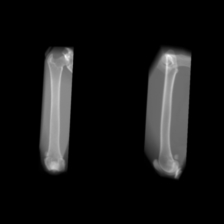

Supplement: Supplementary file 1 — Supplementary Data 1 [file 42003_2020_1057_MOESM1_ESM.zip › 24_bones_classifier_train_valid_224x224x3/train/0/0_APML.dcm_rotation70,-3_energy158.jpg]
