# Supplementary material for: Estimating and abstracting the 3D structure of feline bones using neural networks on X-ray (2D) images
Source: Commun Biol. 2020 Jun 30;3:337. doi: 10.1038/s42003-020-1057-3 (PMC7326932; doi:10.1038/s42003-020-1057-3)
Supplement: Supplementary file 5 — Description of Additional Supplementary Files [file 42003_2020_1057_MOESM5_ESM.pdf]

Description of Additional Supplementary Files

File Name: Supplementary Data 1
Description: Training and validation dataset for “Supplementary Software 1.zip”

File Name : Supplementary Data 2
Description: Embeddings of the images of the 24 bones, as generated by the fully trained Triplet network. Underlying data for the plots in Figure 5.

File Name: Supplementary Software 1
Description: Custom software to pre-process data, train neural networks and classifiers, and to classify images.

File Name: Supplementary Software 2
Description: Pre-trained Triplet neural network and kNN classifier. These files are created as the result of the training process when using the scripts in “Supplementary Software 1.zip”.
