# Supplementary material for: Estimating and abstracting the 3D structure of feline bones using neural networks on X-ray (2D) images
Source: Commun Biol. 2020 Jun 30;3:337. doi: 10.1038/s42003-020-1057-3 (PMC7326932; doi:10.1038/s42003-020-1057-3)
Supplement: Supplementary file 6 — Reporting Summary [file 42003_2020_1057_MOESM6_ESM.pdf]

nature research

Corresponding author(s): Jana Cavojska
Last updated by author(s): May 14, 2020

Reporting Summary

Nature Research wishes to improve the reproducibility of the work that we publish. This form provides structure for consistency and transparency in reporting. For further information on Nature Research policies, see Authors & Referees and the Editorial Policy Checklist.

Statistics

For all statistical analyses, confirm that the following items are present in the figure legend, table legend, main text, or Methods section.

n/a	Confirmed	
☐	☒	The exact sample size (*n*) for each experimental group/condition, given as a discrete number and unit of measurement
☐	☒	A statement on whether measurements were taken from distinct samples or whether the same sample was measured repeatedly
☒	☐	The statistical test(s) used AND whether they are one- or two-sided<br>*Only common tests should be described solely by name; describe more complex techniques in the Methods section.*
☒	☐	A description of all covariates tested
☒	☐	A description of any assumptions or corrections, such as tests of normality and adjustment for multiple comparisons
☐	☒	A full description of the statistical parameters including central tendency (e.g. means) or other basic estimates (e.g. regression coefficient) AND variation (e.g. standard deviation) or associated estimates of uncertainty (e.g. confidence intervals)
☒	☐	For null hypothesis testing, the test statistic (e.g. *F*, *t*, *r*) with confidence intervals, effect sizes, degrees of freedom and *P* value noted<br>*Give P values as exact values whenever suitable.*
☒	☐	For Bayesian analysis, information on the choice of priors and Markov chain Monte Carlo settings
☒	☐	For hierarchical and complex designs, identification of the appropriate level for tests and full reporting of outcomes
☒	☐	Estimates of effect sizes (e.g. Cohen's *d*, Pearson's *r*), indicating how they were calculated

Our web collection on statistics for biologists contains articles on many of the points above.

Software and code

Policy information about availability of computer code

Data collection	2D X-Ray images (Model name: Trixell Pixium 4600), 3D data via CT (Model name: Brilliance 16)
Data analysis	During pre-processing, MeVisLab 3.1.1GCC-5.4.0 to generate artificial 2D X-ray images for each of the CT scans by varying the viewing angle and radiation amount, thus simulating the variability of conditions under which natural X-ray scans are performed.<br>For evaluation purposes, the 3D shape in the STL format was extracted using MeVisLab from each 3D CT scan so that the difference between a predicted shape and the true shape could be measured.<br><br>Deep learning models reported in this work used standard libraries and scripts that are publicly available in TensorFlow 1.8.0 (Google Inc.) and Keras 2.0.8. The custom code for generating triplets and the Triplet network was written in Python 2.7.12. The evaluation, i.e determining the Hausdorff and RMS distance of the 3D models, was performed using MeshLab v2016.12.<br><br>All the software was developed and executed on an Ubuntu 16.04 operating system.

For manuscripts utilizing custom algorithms or software that are central to the research but not yet described in published literature, software must be made available to editors/reviewers. We strongly encourage code deposition in a community repository (e.g. GitHub). See the Nature Research guidelines for submitting code & software for further information.

Data

Policy information about availability of data

All manuscripts must include a data availability statement. This statement should provide the following information, where applicable:

- Accession codes, unique identifiers, or web links for publicly available datasets
- A list of figures that have associated raw data
- A description of any restrictions on data availability

We declare that all the data supporting the findings of this work are available within the manuscript and Supplementary Information files. Raw images can be

requested from the corresponding author.
The instruction how train and use models is provided as supplementary files.

Field-specific reporting

Please select the one below that is the best fit for your research. If you are not sure, read the appropriate sections before making your selection.

☒ Life sciences ☐ Behavioural & social sciences ☐ Ecological, evolutionary & environmental sciences

For a reference copy of the document with all sections, see nature.com/documents/nr-reporting-summary-flat.pdf

Life sciences study design

All studies must disclose on these points even when the disclosure is negative.

Sample size	29 3D CT scans of 29 femurs of different cats. We generated 900 artificial 2D X-ray images from each 3D CT scan (by varying the viewing angle and radiation amount, thus simulating the variability of conditions during X-ray scans), resulting in 26100 2D X-ray like images.
Data exclusions	None.
Replication	All of the Triplet network training experiments to determine the relevance of variables such as margin value, dataset size, specific neural network layers etc. were conducted at least three times and the average validation accuracy was reported in the paper after we have determined that all observed accuracies (for experiments with the same parameters) stayed within the same interval (0.2%).
Randomization	Training, validation and testing images were randomly selected.
Blinding	All the performance testing of the deep neural network results was blindly performed on images that were not included in the training or validation phase of the deep learning method.

Reporting for specific materials, systems and methods

We require information from authors about some types of materials, experimental systems and methods used in many studies. Here, indicate whether each material, system or method listed is relevant to your study. If you are not sure if a list item applies to your research, read the appropriate section before selecting a response.

Materials & experimental systems

n/a	Involved in the study	
☒	☐	Antibodies
☒	☐	Eukaryotic cell lines
☒	☐	Palaeontology
☒	☐	Animals and other organisms
☒	☐	Human research participants
☒	☐	Clinical data

Methods

n/a	Involved in the study	
☒	☐	ChIP-seq
☒	☐	Flow cytometry
☒	☐	MRI-based neuroimaging
